# Testing the Potential of Magnetic Resonance Dosimetry: The Case of Lithium Carbonate

**DOI:** 10.3390/ma18173986

**Published:** 2025-08-26

**Authors:** Alexander Shames, Alexander Panich, Lonia Friedlander, Olga Iliashevsky, Haim Cohen, Raymond Moreh

**Affiliations:** 1 Department of Physics, Ben-Gurion University of the Negev, Beer-Sheva 8410501, Israel; sham@bgu.ac.il (A.S.);; 2 Ilse Katz Institute for Nano-Scale Science and Technology, Ben-Gurion University of the Negev, Beer-Sheva 8410501, Israel; friedlal@bgu.ac.il; 3 Department of Chemistry, Ben-Gurion University of the Negev, Beer-Sheva 8410501, Israel; 4 Department of Chemical Sciences, Faculty of Exact Science, Ariel University, Ariel 40700, Israel; hcohen@ariel.ac.il

**Keywords:** lithium carbonate, gamma radiation dosimetry, electron paramagnetic resonance, nuclear magnetic resonance, paramagnetic defects, nuclear spin–lattice relaxation time

## Abstract

Magnetic resonance techniques are powerful, nondestructive, non-invasive tools with broad applications in radiation dosimetry. Electron paramagnetic resonance (EPR) enables direct quantification of dose-dependent radiation-induced paramagnetic defects, while nuclear magnetic resonance (NMR) reflects the influence of such defects through changes in line width and nuclear spin relaxation. To date, these methods have typically been applied independently. Their combined use to probe radiation damage in the same material offers new opportunities for comprehensive characterization and preferred dosimetry techniques. In this work, we apply both EPR and NMR to investigate radiation damage in lithium carbonate (Li_2_CO_3_). A detailed EPR analysis of γ-irradiated samples shows that the concentration of paramagnetic defects increases with dose, following two distinct linear regimes: 10–100 Gy and 100–1000 Gy. A gradual decay of the EPR signal was observed over 40 days, even under cold storage. In contrast, ^7^Li NMR spectra and spin–lattice relaxation times in Li_2_CO_3_ exhibit negligible sensitivity to radiation doses up to 1000 Gy, while ^1^H NMR results remain inconclusive. Possible mechanisms underlying these contrasting behaviors are discussed.

## 1. Introduction

Measurement of radiation dose, i.e., the amount of energy absorbed by tissue upon ionizing radiation, is crucial for ensuring accurate radiation treatment in medicine, protecting workers from radiation exposure, and understanding the effects of radiation in various fields. Several types of detectors, such as alanine detectors, plastic scintillators, diamond detectors, optical, radio-photoluminescence dosimeters, are used for clinical purposes. They contain materials sensitive to different types of radiation (e.g., gamma-rays, neutrons, electrons, etc.).

Among the various methods, electron paramagnetic resonance (EPR) is a good non-destructive, non-invasive technique based on the measurements of radiation-induced dose-dependent paramagnetic defects [1,2,3,4,5,6,7,8,9,10,11,12,13,14,15,16,17,18,19]. This method has been successfully applied to the study of radiation defects in various materials. For example, such compounds as alanine (C_3_H_7_NO_2_), magnesium and calcium sulfates (MgSO_4_ and CaSO_4_, respectively), lithium carbonate Li_2_CO_3_, and others have been studied for their potential to address this issue [1,2,3,4,5,12,13,14]. Of particular interest are EPR investigations of radiation effects on the human body, such as EPR dosimetry of tooth enamel [6,7,8,9,10,11,15,16,17,18] and stereotactic radiotherapy of the lungs [19]. These studies include dose estimation in tooth enamel using EPR recording for residents near the Chernobyl reactor [6] and variability of radiation doses reconstructed by EPR in teeth of former United States nuclear workers [15].

Among non-biological materials applied in EPR dosimetry, alanine has emerged as the most important and extensively studied system. Since the early 1980s, it has been recognized as a stable and reliable dosimetric medium [4,13,20,21,22,23,24,25,26,27,28,29,30,31,32,33,34,35,36,37,38,39,40,41,42,43,44,45,46,47,48,49,50], suitable for a broad range of applications. Beyond gamma radiation, alanine-based dosimetry has been successfully employed with low-energy X-rays [13], electron beams [20,23,24,25,45], and proton beams [25,29,33,34]. Considerable effort has been devoted to its use in radiation therapy [21,22,27,30,31,39,40,46,47,48,49,50], covering dose levels from relatively low [35,38] to extremely high [48]. A consistent focus across these studies has been on minimizing measurement uncertainties. Factors such as temperature [21,42], humidity [21,44], signal kinetics over time [32], beam-quality dependence [23], and instrument drift [43] have been systematically characterized, with practical correction strategies developed—including adjacent reference standards, standardized sample conditioning, and advanced numerical signal processing [7,15,29,32,34,36].

Neutron and gamma-irradiated lithium carbonate as a material for radiation dosimetry has also been studied using EPR by several authors [12,51,52,53]. Two radicals, CO_2_^−^ with *g*-factor *g* = 2.0006 and CO_3_^−^ with *g* = 2.0036, correspondingly, were reportedly produced as a result of irradiation. The intensities of the ESR signals of these radicals followed a linear dependence on the radiation dose within the dose ranges of 10–800 Gy and 5–800 Gy, respectively. The lowest dose detected by Murali et al. [12] using the signal of CO_2_^−^ was 3.2 Gy. Herewith, Popoca and Urena-Núnez [52] found that the free radicals in the irradiated lithium carbonate have no long-term stability at room temperature; the CO_2_^−^ signal fading was ~12% in the first 5 days and ~55% for 40 days. However, storage of the samples at 0 °C reduced the fading to 10% for 40 days.

An EPR study of neutron-irradiated lithium carbonate in the fluence range 10^12^ to 10^15^ cm^−2^ has been investigated by Herrera et al. [51]. The authors obtained the aforementioned CO_2_^−^ and CO_3_^−^ radicals with g-factors 2.0006 and 2.0036, respectively, and linear dependence of the EPR signal of CO_2_^−^ in the above fluence range. They found a ~15% fading of the CO_2_^−^ signal in the irradiated lithium carbonate stored under ambient conditions and functionally no fading in the sample stored at 0 °C. The detection limit was found to be 5.17 × 10^9^ cm^−2^.

Recently, Rech et al. [53] studied the EPR signal dependence on the irradiation dose and signal stability over 30 days in Li_2_CO_3_ and several other lithium and sodium salts exposed to a 50 kV x-ray source with doses from 5 to 100 Gy. A linear dependence of the signal on irradiation dose was obtained for most compounds including lithium carbonate, while several compounds show exponential dependence on the irradiation dose. The observed signal fading in lithium carbonate was ~40% for 30 days.

EPR dosimetry, while a valuable tool extensively performed with X-band EPR, has certain limitations and shortcomings. Thus, speaking on tooth-based radiation dosimetry, major drawback for emergency assessment purposes is the requirement that the measurements be performed ex vivo, thereby requiring invasive tooth extraction. There is also the possibility of overestimating the dose from surface contamination [21,54]. To overcome the former problem there has been an extensive effort to develop in vivo EPR dosimetry using the L-band (1.2 GHz) [55,56,57]. The L-band is also necessary to reduce the large background microwave absorption due to moisture in the mouth [54].

Following EPR, nuclear magnetic resonance (NMR) was applied to study the radiation dosimetry. NMR measurements show a visible dependence of the nuclear spin–lattice relaxation on the amount of paramagnetic defects created by doping or induced by radiation of a material [58,59,60]. It is worth mentioning that NMR relaxation-based radiation dosimetry has an undeniable advantage over conventional EPR dosimetry: NMR relaxation parameters do not depend on absolute values of magnetic resonance signals’ intensities. Indeed, precise and homogeneous measurements of signals’ intensities are the most problematic issue in EPR dosimetry. Gore et al. [61], Appleby et al. [62], and Schulz et al. [63] investigated the NMR relaxation properties of irradiated Fricke or ferrous sulfate solutions and gels, and showed that radiation-induced changes in these materials, in which ferrous (Fe^2+^) ions are converted to ferric (Fe^3+^) ions, could be quantified using NMR relaxation measurements. Using these and related techniques, Appleby et al. [62] obtained three-dimensional (3D) imaging of spatial radiation dose distribution. Audet et al. [64] and Maryanski et al. [65] reported changes in both NMR transverse and longitudinal relaxation measurements of irradiated polymers, which showed that the relaxation rates increased with the absorbed dose. It was demonstrated that such measurements can be used for dosimetric control of gamma radiation using MRI [61,62,63,64,65,66,67,68,69,70,71,72], i.e., for in vivo MRI dosimetry. Locarno et al. [68] have recently conducted an in-depth ^1^H NMR investigation into the three-dimensional (3D) spatial distribution of radiation dose, which includes doses released in healthy tissues. This work aims to provide clinicians and medical physicists with effective and reliable 3D dosimetric measurements.

Most NMR dosimetric studies were performed using different gels [61,62,63,64,65,66,67,68,69,70,71,72,73,74,75,76]. To our knowledge, NMR studies of solids for this purpose are lacking, and the only known nuclear quadrupole resonance (NQR) investigation of irradiated samples is reported [77]. We therefore present the following study of solid Li_2_CO_3_.

## 2. Materials and Methods

We used lithium carbonate powder Li_2_CO_3_ from Sigma-Aldrich (St. Louis, MO, USA), CAS# 554-13-2, with a purity of ~99%.

XRD measurements were conducted at the Ilse Katz Institute for Nano-scale Science and Technology at Ben-Gurion University of the Negev. The measurements were collected in Bragg–Brentano symmetric reflectance (θ/2θ) geometry using a Panalytical Empyrean III (Malvern Panalytical B.V., Brussels, Belgium) multi-purpose diffractometer equipped with a Cu X-ray source (K_a_ radiation, λ = 1.541Ǻ), operated at *v* = 45 kV, I = 40 mA. The instrument was further equipped with the iCore-dCore automated XRD optics combination, a pixCEL 3D detector in 1D line-detector mode, and an automated spinning stage for back-filled sample holders. The stage can be used in either reflection or transmission mode, and data were collected in reflection mode with a rotation speed of one rotation per second. Collected data were analyzed for phase identification and crystallographic direction by comparison to lithium carbonate diffraction patterns found in the International Center for Diffraction Data (ICDD) Powder Diffraction File (PDF-5+) database (2024). Crystallite size was computed by Reitveld analysis of the whole diffraction pattern using Malvern Panalytical HighScore Plus version 5.1.

All EPR and NMR measurements were performed with as-received samples and those exposed to Co^60^ gamma radiation with the doses ranging from 10 to 1000 Gy. The irradiation of the samples was carried out in a Gammacel 220 with a Co^60^ γ source. The lithium carbonate was irradiated in small glass containers, 15 mL volume, under air atmosphere at a dose rate of 1.85 Gy/minute. The irradiation temperature was 22 °C. Immediately after the irradiation, the containers were stored in a refrigerator at 5 °C until the measurements were conducted. The dose rate was determined using Fricke dosimetry, with a G value of G = 15.6 [78,79].

Continuous wave X-band (ν ~ 9.7 GHz) EPR measurements on initial and gamma-irradiated polycrystalline Li_2_CO_3_ samples were carried out using a Bruker EMX-220 spectrometer (Bruker BioSpin, Rheinstetten, Germany) equipped with an Agilent 53150A frequency counter at room temperature (RT, *T* ~ 295 K). As received and irradiated samples (~100 mg of each) were placed into 5 mm. o.d. Wilmad quartz EPR tubes and centered into the Bruker ER 4102ST TE_102_ cavity for measurements. Then, 40 days after the irradiation, samples (~200 mg of each) were re-measured in 5 mm o.d. standard NMR tubes. Precise determination of *g*-factors (for spin *S* = 1/2 species) and densities of paramagnetic centers *N*_s_ was performed by a comparison with the reference sample—well-purified detonation nanodiamond (DND) powder with *g* = 2.0028(2) and the spins’ content *N*_s_ = 6.3 × 10^19^ spins/g (or 1255 ppm) [80]. Spectra and data processing were performed using Bruker’s WIN-EPR and Origin 7.0 software (OriginLab, Billerica, MA, USA).

RT ^7^Li and ^1^H NMR measurements were carried out in a static mode on a pulse NMR spectrometer Bruker Avance III 500, operating at resonance frequencies 194.37 and 500.13 MHz for ^7^Li and ^1^H, respectively, in an external magnetic field of 11.7467 T. The spectra were measured using the 16-phase cycled spin echo pulse sequence, and the spin–lattice relaxation times *T*_1_ were measured using a saturating comb pulse sequence ($\frac{\pi }{2}$ pulse train) for ^7^Li and inversion recovery pulse sequence for ^1^H. The durations of the $\frac{\pi }{2}$ pulses were 33.3 and 11.6 μs for ^7^Li and ^1^H nuclei, with the amplifiers’ power of 25 and 67.5 W, respectively. Data processing and simulations were performed using the Origin 7.0 software (OriginLab, Billerica, MA, USA).

Note that Herrera et al. [51] and Popoca et al. [52] showed some fading of the EPR signal of irradiated lithium carbonate at room temperature. To slow down this process, we stored the irradiated samples in a fridge at 4 °C.

## 3. Results and Discussion

### 3.1. XRD Data

**Figure 1:**
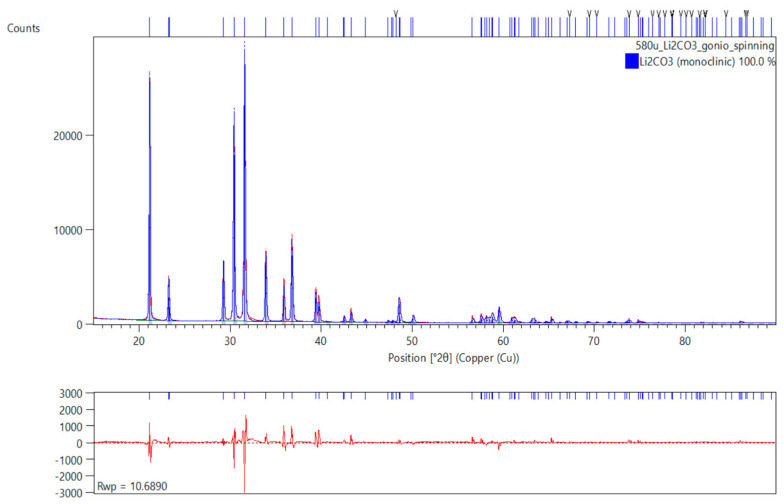
Graphical display of the phase identification and Rietveld analysis result. The red underlying diffractogram in the upper plot is the original raw collected data. The blue overlay is the identified phase result fitted to the raw data by Rietveld refinement. The lower plot shows the difference plot between the fitted phase identification and the raw result. The weighted profile residual (Rwp) value shows a reasonable goodness of fit.

According to the literature, x-ray diffraction (XRD) data [81,82], Li_2_CO_3_ crystallizes in a structure belonging to the monoclinic symmetry, space group $C_{2h}^{6}$-*C2/c*. Unit cell parameters are given as *a* = 8.39(2) Å, *b* = 5.00(1) Å, *c* = 6.21(2) Å, *β* = 114.5(5)°, V = 237.05 Å^3^, Z = 4 [81] and *a* = 8.3593(36) Å, *b* = 4.9725(11) Å, *c* = 6.1975(21) Å, *β* = 114.83(3)°, V = 233.8 Å^3^, Z = 4 [82]. Our XRD measurements (Figure 1) showed comparable unit cell parameters and confirmed that our sample is pure (unhydrated) lithium carbonate Li_2_CO_3_. After Rietveld refinement, the unit cell parameters of the tested sample were determined as *a* = 8.36(3) Å, *b* = 4.97(6) Å, *c* = 6.20(1) Å, *β* = 114.7(3)°, V = 234.38 Å^3^, Z = 4. All lithium atoms are equivalent and occupy *8f* positions in the unit cell. Carbon atoms are in *4e* positions, and two inequivalent oxygens are in 4*e* and 8*f* positions. The sample is well-crystalline with medium–large grains. By Rietveld analysis, the crystallite size is 941.1 ± 10.33 Å. There is a small amount of micro-strain in the sample (0.12 ± 0.00037 strain %). And there are grains with both (002) and (110) preferred orientation.

### 3.2. EPR Data

**Figure 2:**
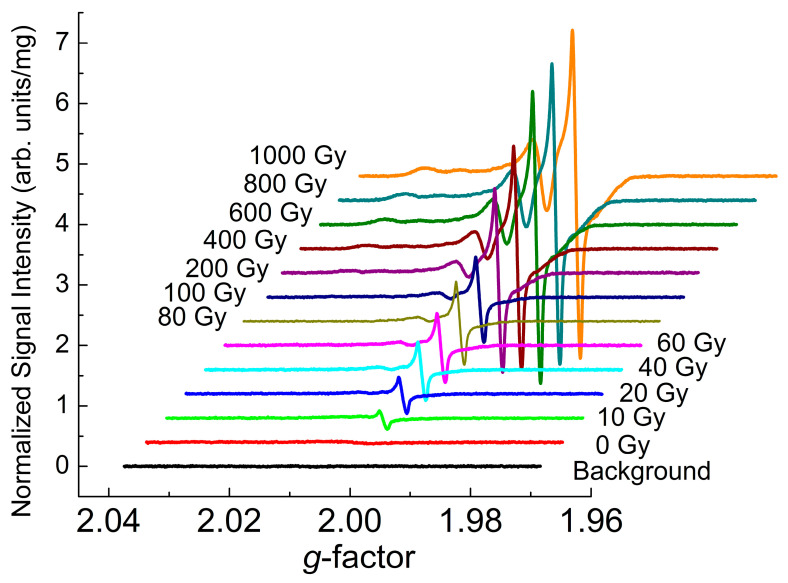
Dose dependence of RT EPR spectra of polycrystalline Li_2_CO_3_ samples registered a few days after the irradiation was performed. The actual irradiation dose is indicated in the figure close to each spectrum. The spectra were registered at non-saturating *P*_MW_ = 2 mW, A_m_ = 0.3 mT, sweep width SW = 12 mT, receiver gain RG = 2 × 10^5^, digital resolution N_P_ = 2048, number of coherent acquisition scans n_aq_ = 100, microwave frequency ν = 9.75 GHz.

The RT EPR spectrum of the initial (as-received) Li_2_CO_3_ sample shows a weak (slightly above the background signal) asymmetric line with *g* ~ 2.003 and line width Δ*H*_pp_ ~ 1.6 mT. Progressive gamma-irradiation causes subsequent growth of clearly observed EPR signals within the range of *g*-factors 2.000–2.026—see Figure 2. It was found that the EPR signals start being saturated at microwave power levels above *P*_MW_ = 5 mW. High resolution spectra (not shown here) reveal the line width of the narrowest component with *g*_iso_ = 2.0007(2) to be Δ*H*_pp_ = 12(2) μT. Aiming to obtain a better signal-to-noise ratio for weaker broad components and, thus, higher reliable quantification of spin contents, all spectra in Figure 2 were further recorded in an over-modulated regime with the 100 kHz modulation amplitude A_m_ = 0.3 mT.

**Figure 3:**
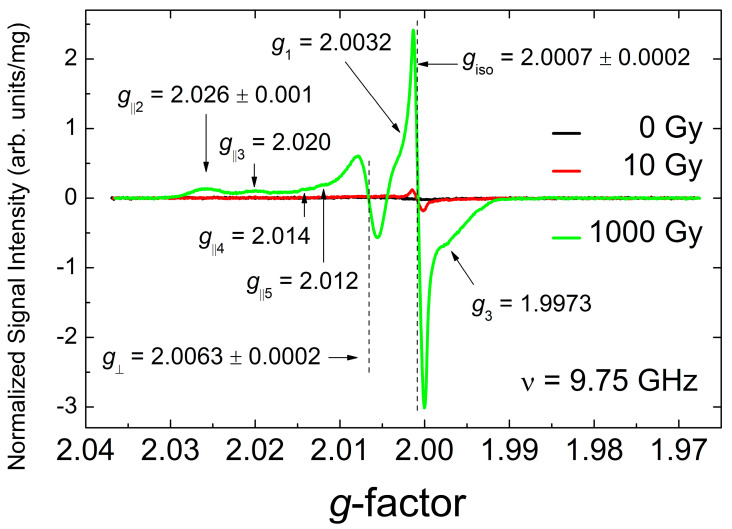
RT EPR spectra of polycrystalline Li_2_CO_3_ samples registered a short time after the irradiation was performed: non-irradiated—black trace, irradiated 10 Gy—red trace, and 1000 Gy—green trace. The spectra were registered at *P*_MW_ = 2 mW, A_m_ = 0.3 mT, SW = 12 mT, RG = 2 × 10^5^, N_P_ = 2048, n_aq_ = 100, ν = 9.75 GHz. Vertical dashed lines and arrows indicate *g*-values found. Background signals have been subtracted.

Figure 3 shows a zoom of the spectra in Figure 2, recorded at the lowest and the highest doses available in the present experiments. At low doses (<20 Gy), the narrow isotropic signal with *g*_iso_ = 2.0007(2) prevails, whereas at higher doses, additional patterns characterized by *g*_⊥_ = 2.0063(2) and various parallel components with 2.026(1) < *g*_||_ < 2.012(1) appears. Here, it is worth mentioning that the higher dose range reveals that the sharp isotropic signal overlaps with a quite intense anisotropic pattern with characteristic *g*-factors *g*_1_ = 2.0032(2), *g*_2_ ~ 2.002(1) (these cannot be determined with higher precision due to being overlapped with the intense narrow signal) and *g*_3_ = 1.9973(2).

**Figure 4:**
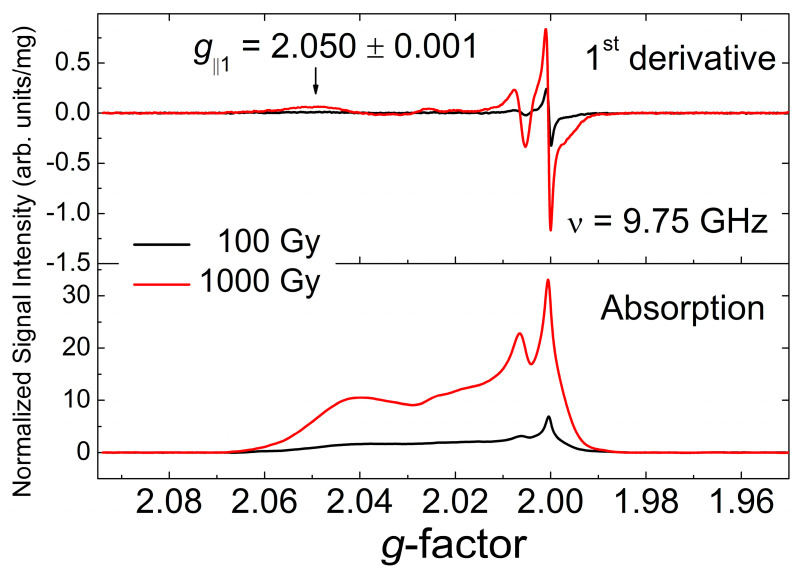
Upper panel: RT EPR spectra of polycrystalline Li_2_CO_3_ samples registered short time after the irradiation: irradiated 100 Gy–black trace, 1000 Gy—red trace. The spectra were registered at *P*_MW_ = 20 mW, A_m_ = 0.1 mT, SW = 30 mT, RG = 2 × 10^5^, N_P_ = 2048, n_aq_ = 49, ν = 9.75 GHz. Arrow indicates additional low-field feature with by *g*_||_ = 2.050(1). Lower panel: absorption EPR spectra obtained by digital integration of EPR spectra at the upper panel. Background signals have been subtracted.

EPR spectra of high-dose irradiated samples recorded at partially saturating high incident power *P*_MW_ = 20 mW reveal an additional low-field feature which is characterized by *g*_||_ = 2.050(1)—see Figure 4. Absorption spectra obtained by digital integration of the conventional 1st derivative EPR signal (Figure 4, upper panel) are presented in Figure 4, lower panel. It can clearly be seen that the paramagnetic species responsible for this component significantly (up to 30%) contributes to the total content of radiation-induced paramagnetic centers in Li_2_CO_3_. The lower panel shows absorption EPR spectra with the background signals subtracted.

**Figure 5:**
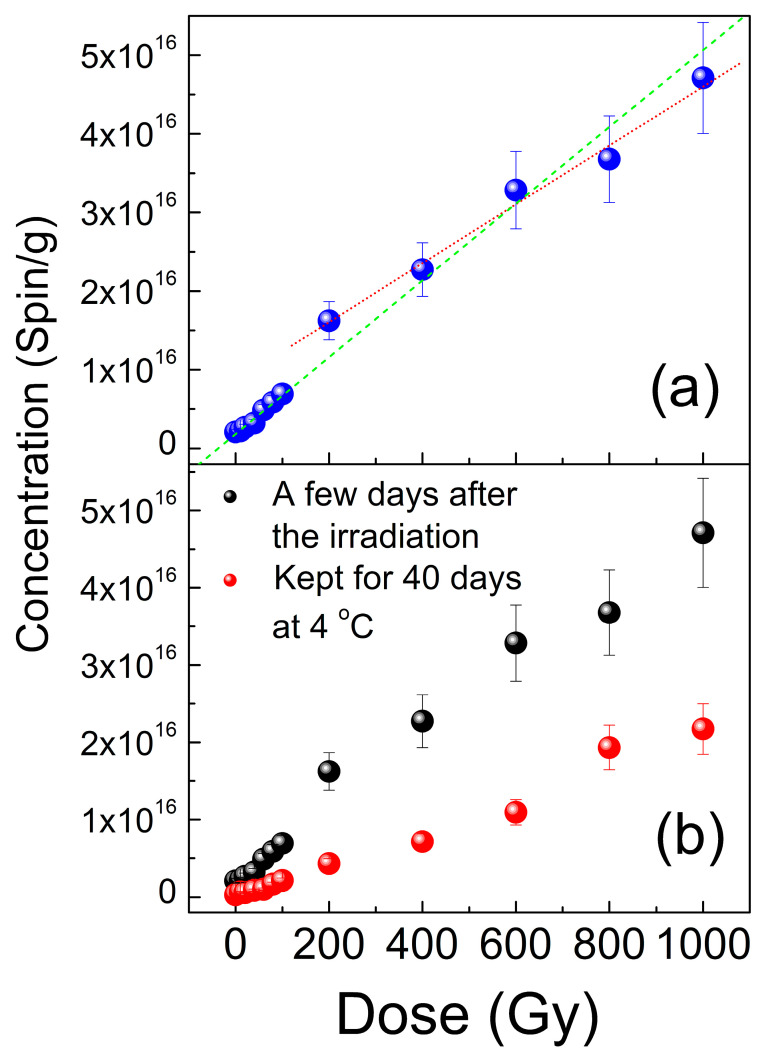
Dependence of the total content of radiation induced paramagnetic defects in Li_2_CO_3_ on gamma irradiation dose: (**a**) results obtained a few days after the irradiation, dashed lines demonstrate linear fits of the dose dependences for the low (green) and high (red) dose regions; (**b**) comparison of contents measured on a series of samples a few days after the irradiation (black symbols) and contents in the same samples kept for 40 days at 4 °C (red symbols).

Figure 5 shows the results of the quantification of the total amount of radiation-induced paramagnetic defects. It may be concluded that the initial sample kept at RT contains a negligible number of paramagnetic centers (~2 × 10^15^ spin/g). In the range of 10–100 Gy, the content of radiation-induced defects increases linearly with dose, reaching ~7 × 10^15^ spin/g. Further irradiation with doses up to 1000 Gy yields a linearly increasing number of paramagnetic defects, reaching ~5 × 10^16^ spin/g at 1000 Gy. Still, the linearity coefficient for this high-dose range appears to differ from that for the low-dose region—see Figure 5a. After the first series of EPR measurements, the samples were placed into NMR tubes, and then a series of ^7^LI and ^1^H NMR experiments was performed. Since the latter required quite a time-consuming procedure, the samples in NMR tubes were kept in the fridge at 4 °C. To assess the stability of radiation-induced centers, all samples were re-measured 40 days after irradiation. It was found that the total content of paramagnetic defects is reduced with time despite keeping the samples at low temperature. In fact, these are the same paramagnetic defects, just as they were found a few days after irradiation (Figure 5b), but the number is reduced. However, the aforementioned quasilinear behavior seems to appear to be retained as well.

Detailed analysis of polycrystalline EPR patterns in Figure 3 and Figure 4 allows for the unambiguous attribution of all paramagnetic defects generated by gamma-irradiation in Li_2_CO_3_ nanopowders. In contrast to the previous publications on irradiated Li_2_CO_3_ [12,51,52] the characteristic intense signal with *g* = 2.0036 attributed there to CO_3_^−^ radicals was never observed in our experiments. Following Refs. [83,84,85,86], the broad anisotropic pattern with factors *g*_1_ = 2.0032(2), *g*_2_ ~2.002(1), *g*_3_ = 1.9973(2) may be reliably identified as originating from CO_2_^−^ radicals in an orthorhombic crystal environment. Attribution of the narrower component with *g*_iso_ = 2.0007(2) in gamma-irradiated Li_2_CO_3_ to the same CO_2_^−^ radicals, proposed in Refs. [12,51,52], raises reasonable doubts. Recently, the isotropic signal with *g*_iso_ = 2.0006 has been attributed to rapidly tumbling CO_2_^−^ radicals associated with occluded water molecules [84,85]. The latter seems to be irrelevant to the case under study. Indeed, the presence of occluded water molecules within the regular Li_2_CO_3_ structure found no justification in our XRD and NMR study (see corresponding sections). Moreover, as was mentioned above, the actual line width of the isotropic *g*_iso_ = 2.0007 line recorded with high resolution (*P*_MW_ = 200 μW, A_m_ = 5 μT, SW = 0.3 mT, RG = 2×10^6^, N_P_ = 2048, n_aq_ = 100) was found to be Δ*H*_pp_ = 12(2) μT, which is at least one order of magnitude smaller than was found in the cases of surrounding occluded water [85] and thermally activated CO_2_^−^ hindered rotations [87]. Thus, it makes sense to propose an alternative attribution of this narrow isotropic line. The most relevant candidates for these paramagnetic defects could be radiation-induced F-centers (electrons trapped at anion vacancies) [88,89] or E’-centers (holes trapped at oxygen vacancies) [90], which are responsible for the observation of narrow lines with *g* < *g*_e_ in gamma-irradiated solids (glasses, quartz, silica). For instance, F-centers in gamma-irradiated sodium-silicate glasses provide sharp line with *g* ~ 2.000 as well as E’-centers in irradiated quartz show signals with *g*_iso_ ~ 2.001.

Another group of radiation induced defects, clearly observed in Li_2_CO_3_, especially at higher doses, is characterized by a complicated polycrystalline pattern which may be characterized by the same (within the current resolution) parallel value of *g*-factor *g*_⊥_ = 2.0063 and multiple *g*_||_ values: *g*_||1_ = 2.050, *g*_||2_ = 2.026, *g*_||3_ = 2.020, *g*_||4_ = 2.014 and *g*_||5_ = 2.012 (Figure 2 and Figure 4). These signals may be attributed to a collection of orthorhombic CO_3_^−^, superoxide O_2_^−^ and O^−^ paramagnetic species, characterized by variably distorted crystalline environments [12,86,90].

### 3.3. NMR Data

After the successful observation of the influence of gamma-irradiation on EPR spectra, we suggested obtaining a similar effect for ^7^Li NMR since nuclear spin–lattice relaxation time (*T*_1_) is known to be sensitive to the presence of the paramagnetic defects.

^7^Li nucleus with spin $I=\frac{3}{2}$ has an electric quadrupole moment, which interacts with the electric field gradient (EFG) created at the nucleus position by the surrounding ions. The Hamiltonian of this interaction is

(1) $$H=H_{z}+H_{Q}+H_{dip}=-\gamma_{n}\hbar B_{0}I_{z}+\frac{e^{2}qQ}{4I(2I-1)}[3I_{z}^{2}-I^{2}+\eta (I_{x}^{2}-I_{y}^{2})]+H_{dip}.$$

**Figure 6:**
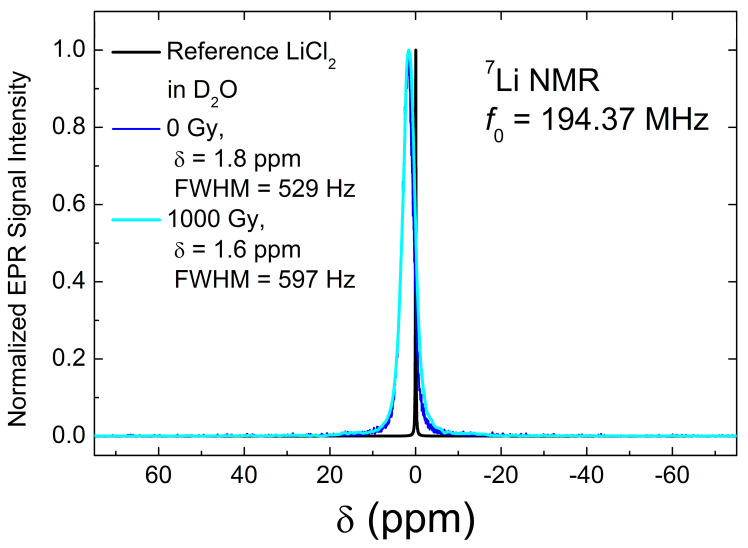
RT ^7^Li spectra in Li_2_CO_3_. Blue trace is the spectrum of the initial as-received sample, and cyan trace is that of irradiated by 1000 Gy. Black trace represents spectrum of the reference LiCl_2_ solution in D_2_O. Line widths and chemical shifts were determined by fitting the spectra to a Gaussian function.

Here, the first term is the nuclear Zeeman interaction with the external magnetic field *B*_0_//z, and the second term describes the quadrupole interaction; *γ*_n_ is the nuclear gyromagnetic factor, *ħ* is the Planck constant, *eq* is the electric field gradient (EFG), *eQ* is the nuclear electric quadrupole moment, and *η* is the asymmetry parameter. The last term represents the dipolar interaction of ^7^Li with the other nuclei; it does not contribute much to the spectra. If *H*_z_ >> *H*_Q_, as in the case of ^7^Li in Li_2_CO_3_ [91], the quadrupole interaction can be treated as a perturbation to the Zeeman interaction. For a $I=\frac{3}{2}$ nucleus, the quadrupolar perturbed NMR spectrum consists of three transitions: the central $\frac{1}{2}\to -\frac{1}{2}$ transition and two satellites $\frac{3}{2}\to \frac{1}{2}$ and $-\frac{1}{2}\to -\frac{3}{2}$ transitions. The latter are shifted to frequencies determined by the product of *ν_Q_* = *e^2^qQ*/*2h* and an angular function related to the orientation of the applied magnetic field in the EFG principal axis frame. In a powder, the satellite lines are distributed over the frequency range of the order of 2ν_Q_ which are difficult to detect. Therefore, the RT ^7^Li spectra in Li_2_CO_3_, shown in Figure 6, are well fitted by a single line. The obtained chemical shift of 1.6–1.8 ppm is characteristic of Li ions that are ionically bound to negatively charged oxygen atoms outside the CO_2_^−^ anions. Some broadening of the spectra, from 529 to 597 Hz, is presumably due to structural defects that arose because of irradiation.

**Figure 7:**
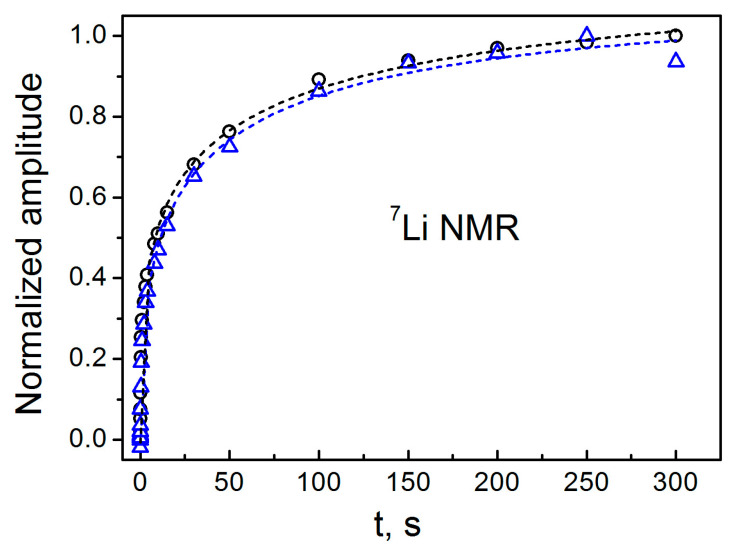
Magnetization recovery in the ^7^Li spin–lattice relaxation (*T*_1_) measurements for the initial Li_2_CO_3_ sample (black circles) and gamma-irradiated with a dose of 1000 Gy (blue triangles). Dashed lines are simulations using stretched exponential functions.

Let us now discuss the spin–lattice relaxation behavior of ^7^Li nucleus in Li_2_CO_3_ shown in Figure 7. The quadrupole coupling shifts the unperturbed Zeeman energy levels, making them unequally spaced, and mixes these states if the EFG is asymmetric. This results in a multi-exponential spin–lattice relaxation behavior [92,93,94]. In most measurements, these exponentials cannot be distinguished from each other. In such a case, the stretched exponential approach is usually used to analyze the multi-exponential relaxation process [95,96,97]:

(2) $$M(t)=M_{\infty }\{1-\exp[-{\left(\frac{t}{T_{1}}\right)}^{\alpha }]\},$$

where $M_{\infty }$ is the equilibrium magnetization, *T*_1_ is the spin–lattice relaxation time, and α is a parameter of exponentiality. The stretched exponential fit of the experimental magnetization recoveries for the initial Li_2_CO_3_ sample and that irradiated with a dose of 1000 Gy is shown in Figure 7. The values of spin–lattice relaxation time before and after irradiation, 40.1 ± 8 and 34.6 ± 7 s, respectively, do not yield a visible difference in *T*_1_ within the experimental errors. (The parameters α obtained in this simulation are 0.37 ± 0.02 and 0.42 ± 0.02, respectively). This means that ^7^Li NMR is not sensitive enough to radiation paramagnetic defects at doses up to 1000 Gy. Such an effect, as well as the rather long spin–lattice relaxation obtained in the experiment, can be explained as follows. In compounds with paramagnetic defects, fast nuclear spin–lattice relaxation is usually driven by spin diffusion [98,99,100,101,102]. This mechanism occurs through the mutual flips of identical neighboring nuclear spins due to interaction terms of $I_{i}^{+}I_{j}^{-}$ type, and results in magnetization transfer from distant nuclear spins to localized electron spins. However, the inequality of the level spacing promoted by the quadrupole coupling excludes the mutual flips of nuclear spins, suppressing spin diffusion, which leads to slow multiexponential spin-lattice relaxation.

Next, although all lithium atoms in lithium carbonate are structurally equivalent, the ^7^Li spins are magnetically non-equivalent due to different directions of the electric field gradients on these nuclei [103]. This obstacle suppresses spin diffusion and slows down relaxation via paramagnetic defects. The paramagnetic defects affect only the closest lithium spins, whose amount is at least five orders of magnitude smaller than the total amount of lithium atoms, and their contribution to relaxation is negligible.

^7^Li spin–spin relaxation measurements do not show visible changes in *T*_2_ for doses of 0 and 1000 Gy, yielding *T*_2_ = 2.33 ± 0.22 and 2.95 ± 0.42 ms, respectively.

**Figure 8:**
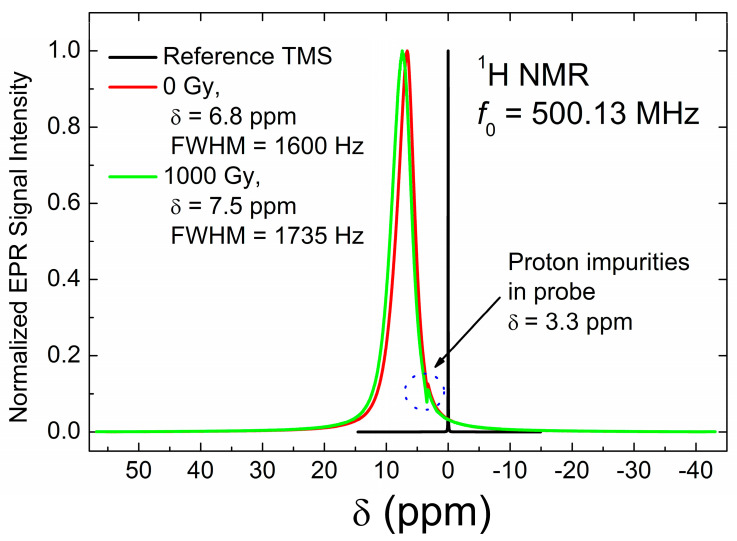
^1^H NMR spectra in Li_2_CO_3_. Red trace—the initial sample, green trace—that irradiated by 1000 Gy. Black trace represents the spectrum of the reference TMS sample. The dotted circle indicates the signal of proton-containing impurities, originating from the NMR probe. Line widths and chemical shifts were determined by fitting the spectra to a Lorentzian function.

**Figure 9:**
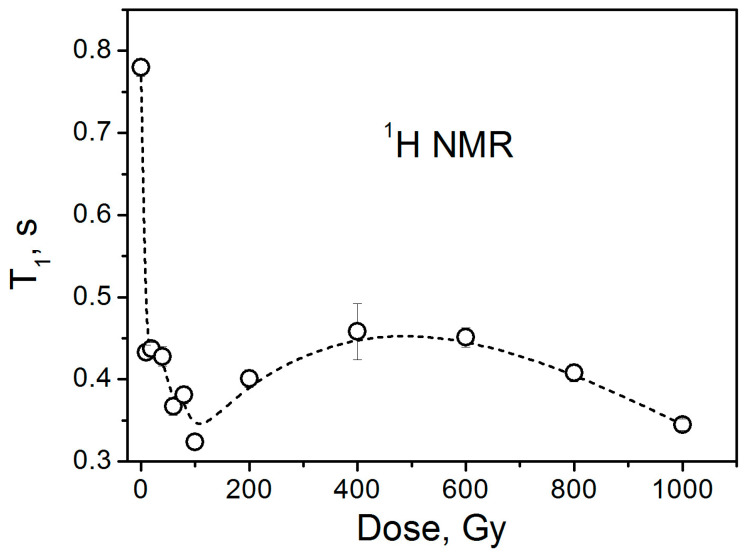
Dose dependence of proton spin–lattice relaxation time.

In addition to ^7^Li, we also measured the ^1^H NMR spectra of the samples under study (Figure 8). The spectra show peaks in the 6.8–7.5 ppm region, which belong to a small number of hydrogen atoms on the surface of the lithium carbonate nanocrystallites, resulting from hydrogen termination due to air exposure during storage. Here, it is worth mentioning that in our ^1^H NMR measurements, no characteristic signals due to water protons (4.7 ppm region) have been observed. This supports the earlier suggestion that the narrow isotropic EPR signal does not originate from rapidly tumbling CO_2_^−^ radicals associated with occluded water molecules. Proton spin–lattice relaxation measurements show a decrease in the relaxation time *T*_1_ with increasing dose up to 100 Gy (Figure 9). Surprisingly, further increases in dose do not lead to a reduction in *T_1H_*. The observed non-monotonic dependence of proton relaxation time on the irradiation dose is not characteristic of nuclear spin–lattice relaxation through paramagnetic centers. We currently have no convincing hypothesis explaining this behavior. Proton spin–spin relaxation (*T_2H_*) measurements do not show visible changes in *T_2H_* for doses of 0 and 1000 Gy.

## 4. Conclusions

In this work, we have conducted a combined EPR and NMR study of radiation defects in the same material for radiation dosimetry. The primary objective of this combined study was to investigate a novel magnetic resonance dosimetry approach that relies on assessing nuclear relaxation parameters in irradiated solids. In contrast to conventional EPR-based dosimetry, this method is designed to circumvent the inherent challenges associated with quantitative EPR measurements.

We studied the application of magnetic resonance techniques—specifically EPR and NMR of ^7^Li and ^1^H—for radiation dosimetry in lithium carbonate (Li_2_CO_3_). A detailed analysis of paramagnetic defects induced by gamma irradiation at doses up to 1000 Gy was performed. The concentration of these defects was found to increase with radiation dose, exhibiting two slightly different linear trends in the ranges of 10–100 Gy and 100–1000 Gy, respectively.

Our measurements also revealed a more than twofold reduction in the EPR signal within 40 days after irradiation, despite the sample being stored in a refrigerator at 4 °C and used only for overnight NMR measurements.

Surprisingly, the nuclear spin–lattice relaxation of ^7^Li nuclei in Li_2_CO_3_ was found to be nearly insensitive to radiation doses up to 1000 Gy, making lithium carbonate unsuitable for ^7^Li NMR-based dosimetry within the tested dose range. An explanation for this behavior is proposed.

## Data Availability

The original contributions presented in this study are included in the article material. Further inquiries can be directed to the corresponding author.
